# Computational Lexical Analysis of the Language Commonly Used to Describe Gout

**DOI:** 10.1002/acr.22746

**Published:** 2016-05-26

**Authors:** N. Lawrence Edwards, Robert Malouf, Fernando Perez‐Ruiz, Pascal Richette, Siobhan Southam, Matthew DiChiara

**Affiliations:** ^1^ University of Florida Gainesville; ^2^ San Diego State University San Diego California; ^3^ Hospital Universitario Cruces and BioCruces Health Research Institute Baracaldo Vizcaya Spain; ^4^ Université Paris Diderot, Sorbonne Paris Cité, and Hôpital Lariboisière Paris France; ^5^ AstraZeneca UK Macclesfield Cheshire, England; ^6^ Ogilvy CommonHealth Behavioral Insights Parsippany New Jersey

## Abstract

**Objective:**

To characterize the current language that is used in describing and defining gout, its symptoms, and its treatment by reviewing recent publications in rheumatology and determining how word choice may, or may not, be reflective of recent scientific developments in gout specifically.

**Methods:**

This was a computational linguistics study, using collocations analyses and concordance analyses on a database of scientific literature related to gout. The final data set for analysis included 2,590 articles, all relating to gout and published between May 2003 and May 2013 and amounting to 12,101,036 tokens (sentence segments). Analysis was conducted by a team of linguists and social scientists.

**Results:**

Our primary finding is that current disease language in gout is marked by ambiguity and imprecision, as evidenced by numerous terms that have similar but distinct meanings, but are nevertheless used interchangeably, therefore blending the slight but significant distinctions between these words. Whereas treatment language is characterized by a multitude of terms to describe a therapeutic mechanism of action, there is a relative void of terms and phrases used to describe success (treating to target) in gout.

**Conclusion:**

The data suggest that the language used to describe gout could be improved and updated. A transformation from an antiquated and insufficiently descript terminological set to one that reflects the recent scientific and clinical advancements made in the category would maximize opportunities for patient and physician understanding.

## INTRODUCTION

Gout is one of the first chronic diseases to be recognized as its own clinical entity, with origins dating to 2640 bc 1, 2. Although scientific and medical understanding of the disease process in gout has inevitably improved since gout's identification, clinicians and documentarians have wavered in the terminology used to refer to the disease. This linguistic ambiguity, or perhaps uncertainty, appears as early as in some of the first recorded uses of the term *gout*. The Latin “gutta quam podagram vel artiticam vocan” translates in English to “the gout that is called podagra or arthritis” 2. This very statement raises the question: is it gout, podagra, or arthritis? Which of these terms, or perhaps what other possibility, most accurately refers to this chronic condition?

Box 1 **Significance & Innovations**

- A team of linguists and rheumatologists collaborated on a comprehensive, computational literature analysis spanning 10 years of scientific and medical literature relating to gout.
- This data‐based study shows that language in gout is characterized by a lack of specificity and consistency.
- Clinical implications are discussed, and recommendations for an improved terminology in gout that would maximize patient adherence and outcomes are made.

Although gout terminology has wavered, the process of scientific discovery and the quest for improved management of gout‐associated pain have necessarily persisted, leading to improvements in its management and treatment 3, 4. The heightened scientific understanding resulting from these inquiries would presumably contribute to a refined disease terminology. Despite the knowledge of the underlying disease process, current disease language emphasizes the flare period of the disease 5. This inadvertently and incorrectly implies that gout is only intermittently active and that the intercritical periods are times when the patient is “disease‐free.” This misperception of clinical symptoms is held by both patients and health care providers, and might explain, at least in part, why gout is so often poorly managed 6. The more correct emphasis should focus on gout as a disease of urate burden that is steadily worsening, even if the symptoms are intermittent, and any language used in defining and describing gout should reflect that reality.

In order to inquire whether or not the language used to describe gout in turn helps or hinders the gout patient, we used computational research to examine the language that is commonly used in the scientific literature to define and describe gout and its effects. Primary areas of analysis included 3 essential categories within health care terminology: disease language, treatment language, and success language.

## MATERIALS AND METHODS

We constructed a representative corpus of primary literature produced by and for medical researchers working on gout. The objective of the article collection was to capture as much language representative of the current state of specialist language in the domain of hyperuricemia as possible; therefore the search process was intentionally designed to be more inclusive than exclusive. Specifically, all available articles that were published in the PubMed Central Open Access Collection (http://www.ncbi.nlm.nih.gov/pmc/tools/openftlist/) between May 2003 and May 2013 and that contained the terms “gout” or “hyperuricemia” were included. The search parameters can be found in Table 1. No exclusions were made based on article type (systematic review, clinical trial, observational study, etc.), country of origin, or publishing journal. The articles in the Open Access Collection are made available by the publishers under a license that allows mass collection and automatic text analysis, and were downloaded as XML files to allow the text to be easily extracted. The initial corpus was then augmented with a collection of 359 manually selected articles from the medical literature that were downloaded as PDF files and converted to text. Each of the manually selected articles matched the same criteria as those from the Open Access subset (listed in Table 1), and were chosen randomly by members of the linguistic team to increase the size of the corpus, therefore providing a more robust data source for analysis.

**Table 1:** Data collection parameters^a^

Parameter	Criteria
Date of publication	May 2003–May 2013
Terms appearing in article	“gout” OR “hyperuricemia”

a The final Boolean search was: (gout OR hyperuricemia) AND 2003/5/1:2013/5/1[Publication Date] AND open access[filter].

Because the corpus consists primarily of articles from the Open Access subset, there may be a concern that it fails to represent the literature in general. Indeed, only about half of the articles matching the search terms are in the Open Access subset and eligible for inclusion in the corpus. Some kinds of researchers may be more likely or less likely to publish in an Open Access venue than others. If we were carrying out a meta‐analysis or other kind of systematic review of previous results, this could be a worrying source of bias. However, in the present work we are not concerned with the scientific content of any of the articles in the corpus or with their results. We are only concerned with the vocabulary used. The size and scope of the corpus, which includes the work of more than 10,000 distinct authors, provides us with a broad picture of the way language is used by the gout research community at large.

**Figure 1:**
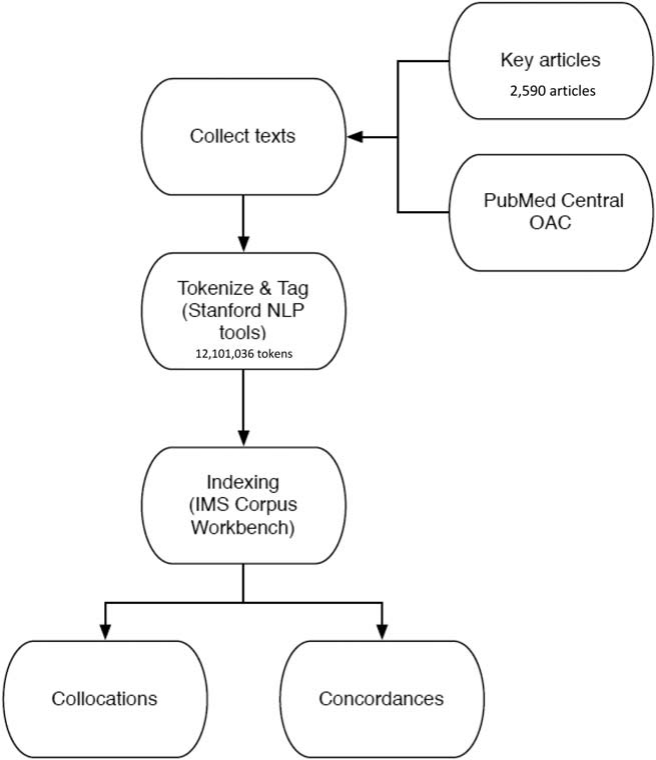
Methodology and process in building and analyzing the corpus. OAC = Open Access Collection; NLP = Natural Language Processing; IMS = Institute für Maschinelle Sprachverabeitung (Institute for Natural Language Processing).

To facilitate further analysis, the combined corpus was next processed using the Stanford Natural Language Processing Group tools, a freely available suite of state‐of‐the‐art utilities for working with natural language text 7. The corpus was tokenized (divided into words and punctuation marks) and then each token was tagged (labeled as to its part of speech or other linguistic function). Finally, the tagged corpus was indexed and stored for later retrieval using the IMS Corpus Workbench 8. Figure 1 illustrates the complete process of building and analyzing the corpus. In all, the corpus contained 2,590 articles with a total of 12,101,036 tokens.

In our analyses of the texts, we primarily used 2 basic techniques. The first was qualitative analysis using a concordance, or an index of all of the occurrences of a word or phrase in the corpus. This allowed us to see the range of contexts in which a word or phrase appears. Since the corpus consists of texts written by and for medical specialists, a concordance provides a snapshot for the analyst of the way that a particular expression is used by authors in this domain.

The second primary mode of analysis was quantitative. Like the concordance analysis, our quantitative approach was based on the premise that words that occur together in texts tend strongly to have related or overlapping meanings. Using the log‐likelihood ratio, a statistical measure of association between words 9, we extracted the collocates of a word of interest, or the words that co‐occurred with the target word at a greater than chance frequency. To find collocates, we first retrieved all occurrences of the target word and then tabulated the frequencies of all of the words (e.g., n = x, where “n” is the term and “x” is the number of times the word appears in the database) that occurred within a 10‐word span to the left or the right of the target. Log‐likelihood scores were then calculated for all of the context words that occurred at least 10 times in context with the target and at least 50 times in the corpus. Context words with the highest scores are the words with the strongest association with the target and are the best indication of the meaning of the target word.

## RESULTS

Using these methods, we analyzed our reference corpus with an emphasis on 3 elements of health care language: disease language (e.g., symptoms, disease names), treatment language (e.g., mechanism of action), and success language (e.g., goals, treatment targets).

#### Disease language

Terms suspected to have the greatest relevance to practicing clinicians were identified, including *gout*, *gouty arthritis*, *hyperuricemia*, *intercritical gout*, *gouty arthropathy*, and *podagra*. Various analyses were conducted on these terms, with particular focus on the term *gout*. Statistically derived collocation analysis of the term *gout* is presented in Table 2. The collocates are words that occur within a 10‐word window of *gout* with much greater than chance frequency. For example, given the overall frequency of *gout* and *flares* in the corpus, one would expect them to co‐occur 61.606 times (on average) in a corpus of this size if they were randomly distributed. In fact, they co‐occur 1,239 times in this corpus, showing a strong association between the 2 words. Strong collocates of *gout* include *flares*, *acute*, *flare*, and *attacks*. These terms frequently co‐occur with *gout*, but are, in actuality, clinically very distinct from it. Based on this analysis, the word that mostly reflects strongly the meaning of *gout* is *flares*, and while this is an accurate term for the physical manifestation of gout, it does not accurately convey the underlying causes of it. In effect, *gout* is used as an umbrella term that refers to the underlying hyperuricemia, the flares and attacks, the acute and chronic stages, and eventually its tophaceous presentation. As a result, this single term, *gout*, conveys at any given time 1 or all of these meanings, including hyperuricemia with crystal deposition, acute gout, intercritical gout, and chronic gout.

**Table 2:** Collocates of the word “gout”

No.	Word	Whole corpus, total no.	Expected collocate frequency	Observed collocate frequency	In no. of texts	Log‐likelihood
1	Flares	1,787	61.606	1,239	140	6,183.611
2	Acute	6,008	207.124	1,983	298	6,025.08
3	Gout	20,869	719.453	3,376	268	5,509.522
4	Hyperuricemia	5,591	192.748	1,799	367	5,364.386
5	Patients	48,092	1657.959	4,712	435	3,964.25
6	Chronic	7,230	249.252	1,670	303	3,827.634
7	Management	3,179	109.595	1,105	210	3,483.311
8	Flare	969	33.406	679	107	3,411.822
9	Tophaceous	613	21.133	548	149	3,281.495
10	Attacks	1,609	55.47	765	202	2,986.124

Further, the phrases *serum urate* and *serum uric* acid were evaluated. Clinically speaking, the majority of the substance in the serum following ionization is the former of these 2, *serum urate*. Data analysis showed more uses of the incorrect term, *serum uric acid* (n = 2,862) than of the correct term, *serum urate* (n = 2,400).

**Figure 2:**
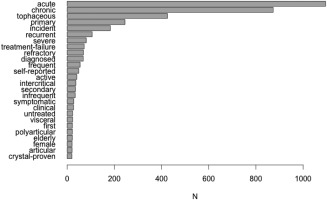
Frequent modifiers of the term gout.

The broad application of and intended meaning for the term *gout* presents other challenges. Many descriptive and qualifying terms that can help clarify and contextualize the intended meaning of another term, including *gouty arthritis*, *symptomatic hyperuricemia*, *intercritical gout*, *monosodium urate crystals*, *tophaceous gout*, *tophus*, *podagra*, *recurrent gout*, *acute gout*, *chronic gout*, *gouty arthropathy*, and *chronic gouty arthritis*, are used. All words occurring as modifiers of *gout* at least 20 times in the database can be seen in Figure 2, and the frequencies of many of the variations in describing *gout* are shown in Table 3. This descriptive nomenclature is applied inconsistently, and therefore obscures the distinctions between words, resulting in the same terms being used to express different meanings.

**Table 3:** Variations in naming and describing gout

Term	Frequency
Gouty arthritis	1,395
Acute gout	1,095
Chronic gout	873
Tophaceous gout	425
Recurrent gout	106
Chronic gouty arthritis	98
Severe gout	76
Gouty arthropathy	44
Intercritical gout	37
Symptomatic hyperuricemia	11
Mild gout	4
Moderate gout	2

Analyzing the term *gout* and how it gets modified reveals that *gout* is used incorrectly to refer to the attack or flare period of the disease. Authors infrequently use the terms *mild gout* (n = 4) and *moderate gout* (n = 2), whereas references to *severe gout* are more frequent (n = 76). The frequency of *severe gout*, combined with the relative void of *mild* or *moderate gout*, shows that the term *gout* is used to refer to the symptomatic manifestation of the disease (i.e., the attack or flare), but might also refer to extension or structural joint damage.

#### Treatment language

The primary goal of therapy in gout, i.e., the decrease in serum urate levels in the bloodstream, can be described using several different terms. *Elimination* (n = 572), *clearance* (n = 2,139), and *excretion* (n = 2,837) are all high‐frequency terms in the database, but with varying degrees of relation to gout. Beyond being used at the greatest frequency overall, the single most common use of *excretion* occurs in the phrase *uric acid excretion* (n = 246), indicating a strong contextual relationship with gout. Although *clearance* is used at similar frequencies to *excretion*, the collocation in which it most commonly appears is *creatinine clearance* (n = 435), suggesting a weaker contextual relationship with gout. *Elimination* is, like *clearance*, not strongly associated with gout. These data suggest, rather unambiguously, that to describe treatment effects, *excretion* is the term that is most accurate and contextually appropriate.

Additional terms relating to therapeutic effect include *dissolution* and *solubility limit*. The latter appeared infrequently (n = 20) in the data set, whereas the former occurred more frequently (n = 263). In the case of *dissolution*, frequent collocates include *crystal*, *crystals*, and *uric*, suggesting a strong contextual relationship to gout.

As an alternative to terminology that conveys a decrease in uric acid, options that convey a balancing of uric acid were also investigated. *Uric acid homeostasis* (n = 9), *uric acid management* (n = 4), *uric acid normalization* (n = 1), and *uric acid inhibition* (n = 1) all demonstrated very low usage rates. Opportunities to evaluate the associative meanings and context in which they are used are therefore limited.

The concept of selectivity in treatments, that a medication may be able to work on specific targets rather than broadly throughout the body, was also evaluated. *Specific*, *targeted*, and *selective* were each analyzed. *Targeted* and *specific* are lower‐frequency terms that are used in a variety of contexts, not limited to gout and uric acid. Comparatively, *selective* occurs more frequently and appears in a pharmacologic context, its most common collocation being *selective inhibitor*.

#### Success language

Despite a multimillion‐word database, success terminology in gout was relatively absent. The terms *control*, *serum uric acid target*, *sUA target*, *serum urate target*, and *treat to target* were each evaluated. *Control* occurs the most frequently, appearing 9,202 times in the data set. However, it is more strongly associated with hyperuricemia than with gout. The phrase *control of hyperuricemia* occurs 116 times in the data set, whereas *uncontrolled gout* (n = 4), *controlled gout* (n = 2), *control of gout* (n = 9), and *gout control* (n = 3) occur very rarely.

Other terms, such as those conveying the concept of treating to a specific goal, or target, were similarly infrequent. *Treat to target* (n = 10), *sUA target* (n = 17), and *serum urate target* (n = 33) occurred rarely, and *serum uric acid target* (n = 0) is a phrase that had never been used in the published literature.

## DISCUSSION

This evidence suggests that gout language is characterized by a lack of specificity and consistency. Similar but not synonymous terms are used in ways that obscure the clinically relevant distinctions between concepts essential to the understanding of gout. For instance, the sheer number of terms that convey similar meanings, e.g., *recurrent gout* and *chronic gout*, *attack* and *flare*, have made it challenging to parse the distinctions between terms, and to apply them in appropriate settings. As evidenced by the common use of modifiers such as *severe* and *moderate* gout and the relative infrequency of the phrase *mild gout*, the term for the very disease that we are evaluating, *gout*, has not remained static either. While its original intended use may have been to refer to the disease itself, the current usage suggests that it has become closely associated with the attack or flare period rather than the underlying disease process, which manifests outwardly as that attack or flare. As it stands, this computational analysis suggests there is no consistently used term to refer to the pathologic process that causes gout symptoms.

Computational data such as these provide insight into the underlying structure of the linguistic system of meaning, and, generally speaking, within the field of computational linguistics, several data points that support the same hypothesis are indicative of a broader finding or implication. While no single data point that we have discovered here is intended to or believed to be the sole reason for comprehensively reforming gout language, these findings individually and collectively warrant a discussion about whether or not gout vocabulary would benefit from revision. The current analysis supports what others working within gout have already suggested, that the language of the category may benefit from an evaluation and revision in light of current clinical knowledge.

A prescription for how to add specificity to our language about gout and help eliminate ambiguity is beyond the scope of this study. We have not addressed these topics in a large enough forum of gout experts, other health care providers, and patients who experience gout to arrive at a consensus. Among the authors there are 3 rheumatologists who are recognized “gout experts.” And although we each have a different native language, we will attempt to start the conversation about adding precision to the language surrounding gout. The comments that follow are the product of thoughtful back‐and‐forth discussion and not from scientifically rigorous surveys. But we believe it is a good place to start.

What should we call this disease that so long ago was called “the gout that is called podagra or arthritis”? Several of us had made a point in our writings over the past 5 to 10 years to refer to it as *gouty arthritis*. Our thinking was that gout is first and foremost an inflammatory arthritis, a basic fact that is lost on most patients with gout. Calling this disease gouty arthritis would formalize this connection and possibly make the painful process more readily understood by patients and health care providers alike. That terminology, however, misses the point that gout is, at its very essence, a metabolic disease of enhanced urate burden with pathologic consequences. Recent advances in imaging have demonstrated sodium urate deposition in joints and periarticular structures long before gout is clinically apparent. This pre‐clinical period may be associated with the same low‐grade inflammation and articular damage (destruction) that we know exists during the intercritical periods of clinical gout. Other consequences of urate deposition may include hypertension and renal and cardiovascular disease. With this understanding, we feel the more generic term *gout* is preferable to the more restrictive term gouty arthritis.

While both *gout flare* and *gout attack* convey a similar sense of escalated pain, the authors feel that these 2 terms may imply a different concept about the underlying disease process. An attack suggests a disease that is only intermittently existent. A flare, on the other hand, connotes an ongoing disease that intermittently worsens. Therefore, *flare* better expresses our current understanding of gout, with its persistent crystal deposition and low‐grade ongoing inflammation that occasionally spirals into an intense cytokine‐driven exacerbation.

In the treatment language of urate‐lowering mechanisms, there is a frequent shuffling of the terms *clearance*, *excretion*, and *elimination*. *Clearance* and *excretion* are terms most often associated with the kidneys’ ability to rid blood of a particular substance. Recently a lot of gout research and drug development has been along the lines of enhancing the kidneys’ ability to rid blood of urate. *Clearance* is a general functional term that describes the amount of a substance filtered out of the blood or the amount of blood cleared of this substance per unit of time. Excretion is a mechanistic term that equals the product of glomerular filtration minus tubular reabsorption plus any tubular secretion. Finally, *elimination* is a term more often linked to organs other than kidneys. The authors, therefore, suggest that is the case of renal handling of urate. The term *excretion* is the most appropriate term. However, at some time in the future, if science and therapeutic design focus on the gastrointestinal tract as a target for urate‐lowering, the term *elimination* may become a more suitable option.

The authors’ final recommendation on how we might refine the language of gout is in the area of treatment success. In many forms of arthritis where new combinations of disease‐modifying antirheumatic drugs and biologic agents make attaining “remission” or “near remission” a realistic goal, we do not refer to this as a cure. This is true for rheumatoid arthritis, psoriatic arthritis, systemic lupus and other connective tissue diseases and vasculitides. This same guarded optimism should be the rule when treating hyperuricemia and gout. We do not cure a metabolic disease by using medication to eliminate the product of the metabolic derangement. While it is true that adherence to therapeutic guidelines for gout will, over time, lead to cessation of signs and symptoms of hyperuricemia and urate crystal deposition, it will not do so irreversibly. We will need to wait for a new era of therapeutic strategies to correct the multiple causes of hyperuricemia before we can truly talk about the cure of gout. The success‐language data reviewed previously do not reflect relative attainability of optimal outcomes. Words or phrases, e.g., *serum urate target*, *serum uric acid target*, and *treat to target*, which convey clinical success in gout, are infrequently used, signifying a general lack of discussion of that which has been deemed clinically achievable (e.g., success in gout).

Rheumatologists have already called attention to the importance of conceptualizing and categorizing clinical aspects of gout 6, and highlighted the need to align on clinical definitions of hyperuricemia 10. Current perceptions of gout, from the general public as well as those from within the health care community, reflect negative, even detrimental, perceptions that can hinder its management and treatment 5. Other illnesses are the priority during clinical training, suboptimal diagnostic techniques are common, long‐term complications are underestimated, treatment lacks proper titration, patient education is suboptimal, and there is low health care practitioner adherence to gout management guidelines 5. Calling for recommendations to the language offers another tool for clinicians and educators to help combat suboptimal outcomes.

In light of recent trends emphasizing the importance of patient health literacy, there is a need to rectify these misperceptions and to foster greater understanding and, therefore, to ultimately improve outcomes. Can improved communication characterized by clearer and more precise language help guide the way? The following evidence suggests yes.

Changes in word choice can produce consequential differences in a hearer's interpreted meaning. When presented with both medical and laypersons' terms referring to the same illness (i.e., erectile dysfunction versus impotence), subjects perceived the medical option to be more serious 11. The importance of patient perceptions regarding their illness and its terminology in relation to their psychological well‐being has also been documented 12.

Simple, understandable language that is related to the physiology of the disease is essential to improving disease management and care. Although clinical obstacles to optimal management and achieving cures in gout have been previously established 10, 13, there is some research demonstrating the role of enhanced communication. Underlining the main components of the disease (causes, risk factors, consequences, and treatment strategies) has been shown to lead to greater adherence to potentially curative gout therapy 14. Recognizing the relationship between language and outcomes, recent guidelines from both the European League Against Rheumatism, as well as the American College of Rheumatology, identify patient education and understanding as core concepts in the care and management of gout 15, 16. Likewise, accurate terminology is a prerequisite for the development of patient‐reported outcomes in gout 17.

Refining medical language, regardless of category, is a daunting challenge with many considerations to be weighed, including clinician preferences, patient preferences, current scientific knowledge of the disease state, the current preferred language of the category, and others. But we argue it is a necessary challenge, and when done correctly can maximize clinical applicability, patient understanding, and ultimately, improve suboptimal outcomes.

## AUTHOR CONTRIBUTIONS

All authors were involved in drafting the article or revising it critically for important intellectual content, and all authors approved the final version to be submitted for publication. Dr. Edwards had full access to all of the data in the study and takes responsibility for the integrity of the data and the accuracy of the data analysis.

**Study conception and design.** Malouf, DiChiara.

**Acquisition of data.** Malouf, DiChiara.

**Analysis and interpretation of data.** Edwards, Malouf, Perez‐Ruiz, Richette, Southam, DiChiara.

## ROLE OF THE STUDY SPONSOR

AstraZeneca had no role in the study design or in the collection, analysis, or interpretation of the data. Publication of this article was contingent upon approval by AstraZeneca.
